# Cardiorespiratory effects of medetomidine and dexmedetomidine combined with tiletamine-zolazepam for the immobilization of Asiatic black bears (*Ursus thibetanus*) under isoflurane general anesthesia

**DOI:** 10.1371/journal.pone.0200833

**Published:** 2018-07-19

**Authors:** Noemi Romagnoli, Giacomo Pagnanelli, Carlotta Lambertini, Emily Drayton, Alessandra Buonacucina, Angelo Peli

**Affiliations:** 1 Department of Veterinary Medical Sciences, University of Bologna, Bologna, Italy; 2 Centralized Veterinary Service, University of Bologna, Bologna, Italy; 3 Chengdu Bear Rescue Center, Sichuan, China; University of Bari, ITALY

## Abstract

The aim of this paper was to compare the cardiorespiratory effects of the two combinations (medetomidine-tiletamine-zolazepam (MTZ) or dexmedetomidine-tiletamine-zolazepam (DTZ)) used for Asiatic black bear (*Ursus thibetanus*) immobilization. A retrospective analysis was carried out, reviewing the anesthetic records of captive bears. Sixty-six records were reviewed. The bears were immobilized, and general anesthesia was maintained with isoflurane vaporized in 100% oxygen. The mean sedation time and score were evaluated. The cardiorespiratory parameters were recorded every 10 minutes from intubation until extubation. Mean sedation time was 26.1 ± 14.5 minutes for the MTZ group and 25.6 ± 19.4 minutes for the DTZ group. The heart rate and the respiratory rate were higher in the bears immobilized with DTZ (66 ± 19 beats/min; 13 ± 5.2 breaths/min) as compared with the bears immobilized with MTZ (57 ± 14.5 beats/min; 10 ± 4.7 breaths/min) whereas the mean arterial pressure did not differ significantly between the groups. The body temperature was in the normal range throughout the procedures in all bears (MTZ 36.3 ± 0.9°C; DTZ 37 ± 1°C). In conclusion, the two protocols used in this study have been proven to be safe and reliable for the immobilization of Asiatic black bears, and the DTZ combination seemed to be associated with less cardiorespiratory depression than the MTZ one.

## Introduction

The Asiatic black bears (*Ursus thibetanus*) are found over a wide area of southern Asia. They occur along the mountains from Afghanistan, through Pakistan and northern India, Nepal, Sikkim, Bhutan, into Burma and north-eastern China. They are also found in south-eastern Russia, and on Taiwan and the Japanese islands of Honshu and Shikoku. Due to the destruction of its habitat and the trade of its body parts, it has been classified as vulnerable by the International Union for Conservation of Nature (IUCN) [1]. The protocols most commonly used for immobilizing bears, include a combination of dissociative anesthetics and benzodiazepines associated with alpha-2 agonists [2–4].

Tiletamine is a dissociative anesthetic commercialized in combination with zolazepam (TZ) in equal volumes. This combination produces rapid and safe immobilization in many species of bears with minimal cardiovascular and respiratory depression [5,6]. The disadvantages of TZ are the lack of visceral analgesia and the absence of a specific antagonist; moreover, additional doses of this combination result in a long recovery period [7]. To reduce these side effects, tiletamine and zolazepam are commonly associated with an alpha-2 agonist in order to immobilize bears [8]. Medetomidine (M) is a highly selective alpha-2 agonist; it is a racemic mixture of two enantiomers: dexmedetomidine and levomedetomidine. The combination of medetomidine-tiletamine-zolazepam (MTZ) has been used in many species of bears [7,9,10], including the Asiatic black bear (*U*. *thibetanus*) [3]. This combination produces deeper anesthesia characterized by better analgesia and muscle relaxation as compared with TZ administered alone [7]. Moreover, the effects of the alpha-2 agonist can be antagonized with the administration of an alpha-2 antagonist. Dexmedetomidine (D), medetomidine’s right enantiomer, is more selective on alpha-2 receptors than the racemic mixture. In brown bears (*Ursus arctos*), the combination of dexmedetomidine-tiletamine-zolazepam (DTZ) induces less hypoventilation and no hypoxia as compared with MTZ [11]. However, Fandos Esteruelas and collegues [10] did not find any differences in respiratory function and partial pressure of oxygen (PaO_2_) when MTZ and DTZ were compared in immobilizing brown bears (*Ursus arctos*).

The effects of the administration of M in combination with TZ for the immobilization of the Asiatic black bear (*U*. *thibetanus*) has already been described [3]; however, no data have been reported regarding the use of dexmedetomidine in the same species. The aim of this study was to compare the cardiovascular and respiratory effects of MTZ and DTZ when used to immobilize Asiatic black bears (*U*. *thibetanus*). The Authors hypothesized that DTZ induced less cardiovascular and respiratory depression as compared to MTZ.

## Materials and methods

### Animals

A retrospective analysis was carried out which reviewed the anesthetic records of captive Asiatic Black bears (*U*. *tibethanus*) undergoing anesthesia for elective clinical assessment between 2013 and 2015. The study was carried out at the Chengdu Bear Rescue Centre, in Sichuan, China. Only records from adult animals (age >1 year) and with an assigned American Society of Anesthesiologists (ASA) status 1 to 2 were considered.

Bears included in the study were rescued by the Animals Asia foundation and housed in appropriate sized cages with environmental enrichments in accordance with standard operating procedures.

### Anesthetic protocol

The anesthetic records were divided into two groups on the basis of premedication which consisted of:

- medetomidine (0.0171 mg/kg) in association with tiletamine (0.17 mg/kg) and zolazepam (0.17 mg/kg) (medetomidine tiletamine zolazepam-MTZ- group)
- dexmedetomidine (0.0081 mg/kg) in association with tiletamine (0.17 mg/kg) and zolazepam (0.17 mg/kg) (dexmedetomidine tiletamine zolazepam-DTZ- group)

Food, but not water, was withheld 12 hours before the procedure. The anesthetic solutions were prepared by dissolving a vial containing powdered tiletamine (250 mg) and zolazepam (250 mg) (Zoletil 100, Virbac, Bury St Edmonds, Suffolk, England) in 5 ml of dexmedetomidine (Dexdomitor, 0.5 mg/ml, Zoetis, New Jersey, U.S.A.) or medetomidine (Domitor, 1 mg/ml, Zoetis, London, England). The anesthetic solution was administered in the brachial triceps muscle using a jab stick syringe (JorVet safety stick pole syringe, Jorgensen Labs, Loveland, Colorado, USA). After the intramuscular (IM) injection, the sedation was evaluated every 5 minutes based on the sedation score which is summarized in Table 1. Sedation time was defined as the time between the IM injection and the moment at which the animals reached a sedation score of three; the duration of the anesthesia was calculated as the time interval between the dart injection and the atipamezole administration.

**Table 1:** Sedation score.

Score	Description
**0**	No effects
**1**	Voluntary movements still present
**2**	Unconscious, rhythmic breath, reflexes still present
**3**	No reflexes, stage of surgical anaesthesia
**4**	Cardiopulmonary arrest
**5**	Death

Sedation score applied for determination of the quality of sedation of Asiatic black bears (*U*. *tibethanus*) undergoing general anaesthesia for clinical evaluations.

Once the surgical anesthetic plane was reached (sedation score three), the bears were placed on the surgical table, brought into the operating room and then positioned in sternal recumbency. In all bears, an 18 G venous catheter was placed in the cephalic vein and fluid therapy (saline solution 0.9% 5 ml kg^-1^ h^-1^) was administered throughout the procedure. Intubation was achieved with animal in sternal recumbency using a 16–18 mm cuffed endotracheal tube under direct visualization of a miller laryngoscope.

General anesthesia was maintained with isoflurane (Isoflo, Abbot, Maidenhead, Berkshire, England) vaporized in 100% oxygen and was delivered through a circle breathing system. All monitoring devices were attached (Midmark TidalGuard HD, 8009-001/002, Midmark Corporation, Ohio, U.S.A) soon after the anesthetic was administered. Briefly, the heart rate (HR) and the arterial oxygen saturation (SpO_2_) were monitored using pulse oximetry (placing the probe on the tongue); the respiratory rate (*f*_R_) and the fraction of expired CO_2_ (Fe’CO_2_) were monitored with a sidestream probe. Body temperature was continuously checked using a rectal thermometer. Systolic (SAP), diastolic (DAP) and mean (MAP) arterial blood pressure were measured using an oscillometric technique by means of an appropriately sized cuff placed on the forelimb (Midmark Cardell Model 9401, Midmark Corporation, Ohio, U.S.A).

Cardiorespiratory parameters, as well as anesthetic depth, based on the evaluation of the palpebral reflex and jaw tone, were recorded every 10 minutes from intubation (T0) until extubation.

At the end of the procedure, the bears recovered in their cages; 5 mg atipamezole (Antisedan, 5 mg/ml, Zoetis, New Jersey, U.S.A) for each mg of medetomidine and 10 mg of atipamezole for each mg of dexmedetomidine were administered intramuscularly. After atipamezole administration, the endotracheal tube was removed.

### Statistical analysis

Data were tested for normality using the Shapiro-Wilk test. Normally distributed data (HR, *f*_R_ MAP) were compared between the groups using the one-way ANOVA test; p<0.05 was considered statistically significant. Normally distributed data are presented as means and standard deviations (SDs). Non-parametric data and scores from sedation are presented as medians and ranges. Weights and anesthesia time were compared using an independent sample t-test; p<0.05 indicated a statistically significant difference. The same test was used to compare HR and *f*_R_ at each time point. MedCalc computer software was used for the data evaluation (version 15.8).

## Results

A total of 66 anesthetic records were considered. Demographic data and type of clinical procedure performed are reported in Table 2. No differences were recorded when comparing the weights of the two groups of animals.

**Table 2:** Demographic data, anaesthesia time and type of procedures for 66 bears (*Ursus tibethanus*) undergoing general anaesthesia.

	MTZ	DTZ
**Number**	45	21
**Weight (kg)**	135 ± 29 (88–218)	146 ± 29 (106–210)
**Sex**		
**Male (n = )**	12	6
**Female (n = )**	33	15
**Anaesthesia time (min)**	120 ± 52 (50–295)	105 ± 32 (65–165)
**Procedure**		
**Health Check (n = )**	15	6
**Dental (n = )**	22	5
**Radiography (n = )**	2	3
**Wound care (n = )**	2	0
**Endoscopy (n = )**	1	0
**Ophthalmology (n = )**	3	7

Before general anaesthesia bears received medetomidine (0.0171 mg/kg) in association with tiletamine (0.17 mg/kg) and zolazepam (0.17 mg/kg) (MTZ- group) or dexmedetomidine (0.0081 mg/kg) in association with tiletamine (0.17 mg/kg) and zolazepam (0.17 mg/kg) (DTZ- group). Values of weight and anaesthesia time are presented as mean ± SD and range

The mean sedation time necessary to reach a sedation score of 3 was 26.1± 14.5 minutes for the MTZ group and 25.6 ± 19.4 minutes for the DTZ group. The anesthesia time did not differ significantly between the two groups.

Some bears required multiple injections to achieve a sedation score of 3. The range of dosages administered were 0.0121–0.0285 mg/kg and 0.0062–0.0141 mg/kg for medetomidine and dexmedetomidine, respectively. The dose range for tiletamine-zolazepam was 0.12–0.28 mg/kg for both the MTZ and the DTZ groups.

**Fig 1:**
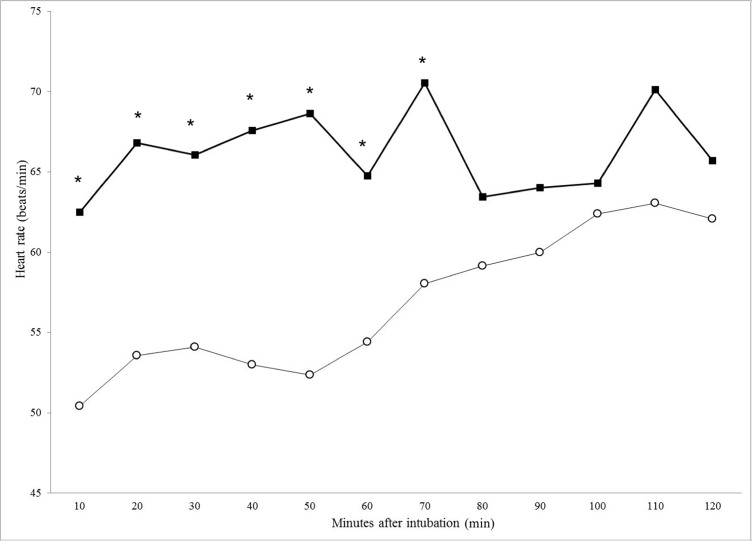
Heart rate recorded at 10 minute intervals in 66 bears (*Ursus tibethanus*) under general anaesthesia. MTZ group (○) received medetomidine (0.0171 mg/kg) in association with tiletamine (0.17 mg/kg) and zolazepam (0.17 mg/kg). DTZ group (■) received dexmedetomidine (0.0081 mg/kg) in association with tiletamine (0.17 mg/kg) and zolazepam (0.17 mg/kg).

The cardiorespiratory parameters recorded under general anesthesia are summarized in Table 3. The HR was higher in the DTZ group at almost all the time points as compared with the MTZ group (Fig 1). Statistically significant differences were observed from T10 up to T70 (p = 0.006, p = 0.004, p = 0.006, p = 0.001, p< 0.001, p = 0.017 and p = 0.022, respectively).

**Table 3:** Cardiorespiratory and anesthetic parameters recorded under general anaesthesia in 66 bears (*Ursus tibethanus*).

	MTZ	DTZ
**Isoflurane (%)**	2.3 ± 0.8	2.6 ± 0.8
**Fe’CO**_**2**_ **(mmHg)**	46 ± 26.7	43 ± 6.3
**SpO₂ (%)**	96 ± 1.6	96 ± 2.2
**HR (beats/min)**	57± 14.5 *	66 ± 19 *
***f*R (breaths/min)**	10 ± 4.7 *	13 ± 5.2 *
**MAP (mmHg)**	104 ± 30.8	105 ± 32.8
**Body temperature (°C)**	36.3±0.9	37±1

Bears received medetomidine-tiletamine-zolazepam (group MTZ—45 bears) or dexmedetomidine-tiletamine-zolazepam (group DTZ—21 bears). The fraction of expired CO_2_ (Fe’CO_2_), hemoglobin saturation (SpO_2_), heart rate (HR), respiratory rate (*f*_R_), mean arterial pressure (MAP) and body temperature are reported. Data are reported as mean ± standard deviation. *: significantly different between the groups (p< 0.05).

**Fig 2:**
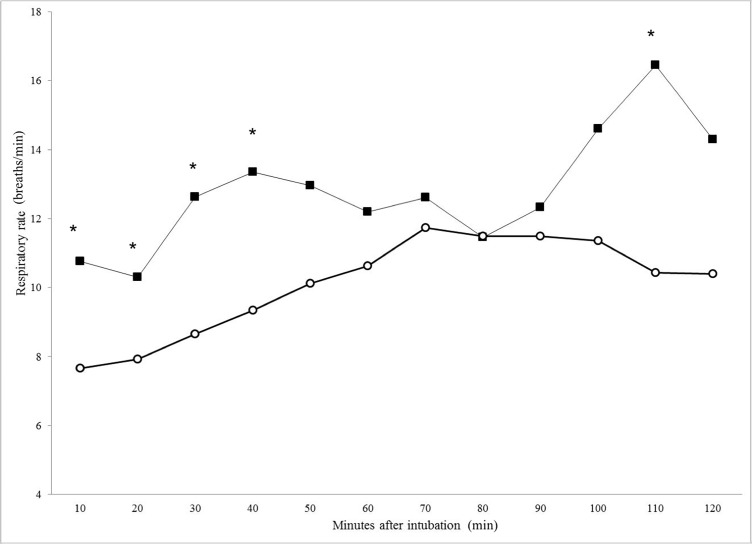
Respiratory rate recorded at 10 minute intervals in 66 bears (*Ursus tibethanus*) under general anaesthesia. MTZ group (○) received medetomidine (0.0171 mg/kg) in association with tiletamine (0.17 mg/kg) and zolazepam (0.17 mg kg). DTZ group (■) received dexmedetomidine (0.0081 mg/kg) in association with tiletamine (0.17 mg/kg) and zolazepam (0.17 mg/kg).

**Fig 3:**
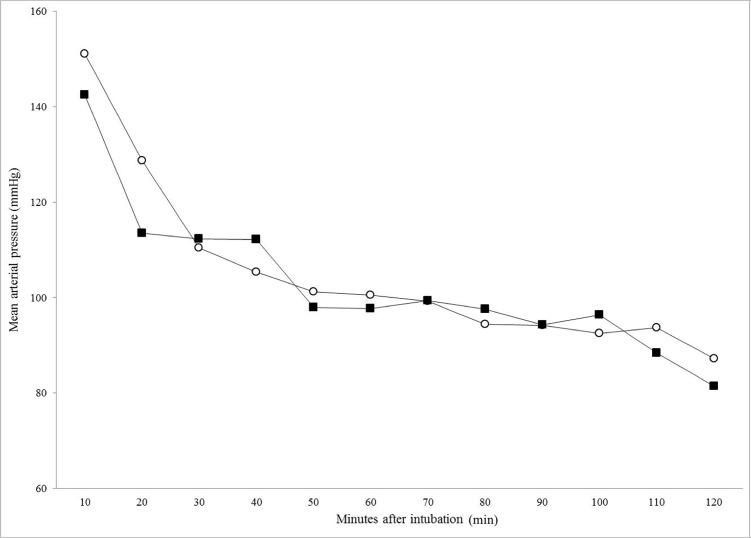
Mean arterial pressure recorded at 10 minute intervals in 66 bears (*Ursus tibethanus*) under general anaesthesia. MTZ group (○) received medetomidine (0.0171 mg/kg) in association with tiletamine (0.17 mg/kg) and zolazepam (0.17 mg/kg). DTZ group (■) received dexmedetomidine (0.0081 mg/kg) in association with tiletamine (0.17 mg/kg) and zolazepam (0.17 mg/kg).

The *f*_R_ was higher in bears treated with dexmedetomidine with respect to those treated with medetomidine (Fig 2). Only from T10 to T40 and at T110 were the differences in the *f*_R_ statistically significant (p = 0.009, p = 0.021, p = 0.001, p = 0.006 and p = 0.005, respectively). The MAP had the same trend in both groups, decreasing over time during the procedure from T10 to T60 and then remaining stable up to T120 (Fig 3). The MAP did not differ statistically between the groups at any time point.

## Discussion

The two protocols used in this study have been proven to be safe and reliable for the immobilization of Asiatic black bears (*U*. *thibetanus*).

A common adverse effect reported during anesthesia with medetomidine in bears is vomiting [12]. No bears in this study vomited or showed sign of vomiting. The advantages of combining alpha-2 agonist with tiletamine and zolazepam are a smooth and rapid induction and a smaller induction dose as previously reported in several papers [6,9,10].

In the present study, the mean induction time in both groups was longer than that reported for other bear species [3,10]. In these previous reports, the anesthetized bears were free-ranging animals which were pursued with a helicopter. The effect of the anesthetic protocol is affected by the teleanesthetic technique applied. When free-ranging animals are captured using helicopter pursuit, the stress and fear felt by the animals result in increased HR, blood pressure and body temperature which alter the metabolism and distribution of the drugs. In the present study, all the anaesthetized bears were confined to a transportation cage which the bear had been desensitised to and trained to enter through operant training techniques.

Alpha-2 agonist administration in different bear species is usually associated with a decrease in HR and a subsequent decrease in cardiac output and blood pressure. A significant decrease in HR has been reported in polar bears (*Ursus maritimus*) and brown bears (*Ursus arctos*) receiving medetomidine in combination with tiletamine-zolazepam [7,9]. Conversely, in black bears (*Ursus americanus*) sedated with dexmedetomidine in combination with tiletamine-zolazepam, the HR remained in the normal range (60–90 beats/min), and tachycardia or bradycardia were not observed [13]. In the present study, the HR was within the normal range in both groups throughout the procedures; however, the mean HR was higher in the DTZ group (66 ± 19 beats/min) as compared with the MTZ group (57 ± 14.5 beats/min) (p<0.001). In addition, in our study, general anesthesia was maintained with isoflurane in both groups. As previously reported in Asiatic black bears (*U*. *thibetanus*) immobilized with medetomidine and tiletamine-zolazepam, the isoflurane produced a dose-dependent increase in HR as well as in *f*_R_ and Fe’CO_2_ [3]. Medetomidine or dexmedetomidine administration has been described to produce a biphasic pressure response in several species. Initial hypertension is frequently observed, followed by hypotension which is sustained by dose-dependent halogenated vasodilation. In our records, blood pressure decreased during the procedure (from T10 to T60) but remained in the normal range, and the mean values did not differ significantly between the groups.

Respiratory depression, characterized by decreased *f*_R_ and hypoxia have frequently been described in bears after pharmacological immobilization [9,14]. In the present study, hemoglobin saturation remained stable, over 91% and the respiratory rate was higher in the DTZ group as compared with the MTZ group. This is in accordance with the study of Teisberg and collegues [15] in which the dexmedetomidine (0.0101 mg/kg IM) associated with tiletamine-zolazepem used for immobilizing grizzly bears (*Ursus arctos*) did not produce hypoxia or any instances of hypoventilation. A limitation of our study is that a blood gas evaluation was not carried out to monitor the PaO_2_. In addition, hemoglobin oxygen saturation was measured only with a pulse oximeter which has been described to be an unreliable indicator of hypoxemia in bears [10,16]. Another important finding during the capture of free-ranging bears was the alteration in body temperature; hyperthermia or hypothermia have been reported especially during anesthesia with helicopter capture [3,9,10,15]. In our study, the rectal temperature was in the normal range throughout the procedures.

One of the advantages of combining an alpha-2 agonist with tiletamine-zolazepam was the possibility of antagonizing the sedative drug with atipamezole. In both groups (MTZ and DTZ) in the present study, the injection of atipamezole at the end of the procedures allowed fast recovery in all animals as has previously been reported by several authors for different bear species [3,9,10,13]. In conclusion, this retrospective study demonstrated that medetomidine and dexmedetomidine combined with tiletamine-zolazepam provided smooth and effective immobilization in captive Asiatic black bears (*U*. *thibetanus*). The DTZ combination was associated with less cardiorespiratory depression as compared with the MTZ combination. No side effects were observed in the animals included.

Additional studies are necessary to investigate the efficacy and safety of these protocols in sick animals, ASA status 3 and 4.
